# Mapping the Risk of Snakebite in Sri Lanka - A National Survey with Geospatial Analysis

**DOI:** 10.1371/journal.pntd.0004813

**Published:** 2016-07-08

**Authors:** Dileepa Senajith Ediriweera, Anuradhani Kasturiratne, Arunasalam Pathmeswaran, Nipul Kithsiri Gunawardena, Buddhika Asiri Wijayawickrama, Shaluka Francis Jayamanne, Geoffrey Kennedy Isbister, Andrew Dawson, Emanuele Giorgi, Peter John Diggle, David Griffith Lalloo, Hithanadura Janaka de Silva

**Affiliations:** 1 ICT Centre, Faculty of Medicine, University of Kelaniya, Ragama, Sri Lanka; 2 Department of Public Health, Faculty of Medicine, University of Kelaniya, Ragama, Sri Lanka; 3 Department of Parasitology, University of Kelaniya, Ragama, Sri Lanka; 4 Department of Medicine, Faculty of Medicine, University of Kelaniya, Ragama, Sri Lanka; 5 Clinical Toxicology Research Group, University of Newcastle, Waratah, Australia; 6 South Asian Clinical Toxicology Research Collaboration, University of Peradeniya, Peradeniya, Sri Lanka; 7 Central Clinical School, Faculty of Medicine, University of Sydney, Sydney, Australia; 8 CHICAS, Lancaster University Medical School, Lancaster, United Kingdom; 9 Liverpool School of Tropical Medicine, Liverpool, United Kingdom; Swiss Tropical and Public Health Institute, SWITZERLAND

## Abstract

**Background:**

There is a paucity of robust epidemiological data on snakebite, and data available from hospitals and localized or time-limited surveys have major limitations. No study has investigated the incidence of snakebite across a whole country. We undertook a community-based national survey and model based geostatistics to determine incidence, envenoming, mortality and geographical pattern of snakebite in Sri Lanka.

**Methodology/Principal Findings:**

The survey was designed to sample a population distributed equally among the nine provinces of the country. The number of data collection clusters was divided among districts in proportion to their population. Within districts clusters were randomly selected. Population based incidence of snakebite and significant envenoming were estimated. Model-based geostatistics was used to develop snakebite risk maps for Sri Lanka. 1118 of the total of 14022 GN divisions with a population of 165665 (0.8%of the country’s population) were surveyed. The crude overall community incidence of snakebite, envenoming and mortality were 398 (95% CI: 356–441), 151 (130–173) and 2.3 (0.2–4.4) per 100000 population, respectively. Risk maps showed wide variation in incidence within the country, and snakebite hotspots and cold spots were determined by considering the probability of exceeding the national incidence.

**Conclusions/Significance:**

This study provides community based incidence rates of snakebite and envenoming for Sri Lanka. The within-country spatial variation of bites can inform healthcare decision making and highlights the limitations associated with estimates of incidence from hospital data or localized surveys. Our methods are replicable, and these models can be adapted to other geographic regions after re-estimating spatial covariance parameters for the particular region.

## Author Summary

Snakebite is a neglected tropical disease which mainly affects the rural poor in tropical countries. There is little reliable data on snakebite, which makes it difficult to estimate the true disease burden. Hospital statistics underestimate numbers of snakebites because a significant proportion of victims in tropical countries seek traditional treatments. On the other hand, time limited or localized surveys may be inaccurate as they may underestimate or overestimate numbers depending on when and where they are performed. To get a truer picture of the situation in Sri Lanka, where snakebites are an important cause of hospital admission, we undertook an island-wide community survey to determine the number of bites, envenomings and deaths due to snakebite in the previous 12 months. We found that there were more than 80,000 bites, 30,000 envenomings and 400 deaths due to snakebite, much more than claimed by official statistics. There was variation in numbers of bites and envenomings in different parts of the country and, using the data from our survey, we were able develop snakebite risk maps to identify snakebite hotspots and cold spots in the country. These maps would be useful for healthcare decision makers to allocate resources to manage snakebite in the country. We used free and open source software and replicable methods, which we believe can be adopted to other regions where snakebite is a public health problem.

## Introduction

Bites of venomous snakes cause significant morbidity and mortality in the rural tropics. Despite this, snakebite did not, until very recently, receive the attention it deserves as an important public health problem. The main reason for this was the paucity of robust epidemiological data on the disease burden associated with snakebite. The most recent estimates of the global burden of snakebite highlighted the need for good quality data on snakebite, particularly from nation-wide population-based studies [1].

The paucity of reliable data is partly related to inherent methodological difficulties, which include: poorly developed reporting and recording systems in countries with the highest burden, limitations in hospital-based data that often under-estimate the problem [2][3][4][5], and seasonal and geographical variation in bite incidence [6][7], all of which make extrapolations unreliable. Sound epidemiological data are, however, important to both give credence to the magnitude of the problem and raise awareness of snakebite as an important but neglected public health issue, and to assist prioritization of resources for prevention and treatment.

Sri Lanka has a mean elevation of 228 meters and consists of mainly low flat to rolling plains with mountains in the south central interior which reach 2400 meters. The country is divided into three climatic zones based on rainfall: wet (south western and central parts of the country including the Western, Central and Sabaragamuwa provinces); dry (northern and south eastern parts of the country including the Northern, Northcentral, Eastern and parts of the Southern and Uva provinces); and intermediate (the areas in between the wet and dry zones including the Northwestern, and parts of Uva and Southern provinces) [8][9]. The differences in rainfall have led to much diversity in the flora and fauna, and in land use in these zones, leading to differences in snakebite patterns [1]. Despite being a country where snakebite results in over 30000 hospital admissions annually [10], the community incidence of snakebite in Sri Lanka is unknown. Previous research has shown that hospital data underestimate deaths due to snakebites by over 50% [4], and multiple hospital admissions at different levels of care can further distort hospital statistics.

Geostatistical modeling provides a method to estimate continuous spatial variation in incidence from community level surveys through modeling the occurrence of spatially correlated events in order to predict the probability or likelihood of occurrence of an event anywhere in the study-region and to identify risk-factors [11][12]. The resulting risk maps can provide useful information for healthcare decision making in resource limited settings; they include the majority of the most snakebite endemic regions, without the need for comprehensive registry data.

The aims of this study were to determine the community incidence, rate of envenoming, mortality and geographical pattern of snakebite in Sri Lanka and to develop a snakebite risk map for Sri Lanka based on geo-spatial and socio-demographic predictive factors.

## Methods

### Data sources

#### Epidemiological data

We performed a country-wide community based cross sectional survey between August 2012 and June 2013. The survey was designed to sample approximately 1% of the population of the country. Sri Lanka has nine provinces that encompass 25 districts. The sample was distributed equally among the nine provinces, as our objectives included estimation of incidence for each of the provinces. Based on the estimated population of 18.8 million and 3.9 million households in 18 districts in 2001, we estimated that in 2012 approximately 20 million people live in 4.5 million households in Sri Lanka (Department of Census and Statistics, Sri Lanka, 2001). We therefore planned to sample 45000 households, consisting of 5000 households from each of the country’s nine provinces.

A *GramaNiladhari* (GN) division (the smallest administrative unit in the country), of which there are 14022 in the country, was defined as a cluster for data collection. A multi-stage sampling strategy was adopted. The first stage sampling unit (e.g. clusters) was the GN division. 125 clusters were selected from each of the 9 provinces of Sri Lanka. The number of clusters per district was chosen based on probability proportional to district size. Within each district, the required number of clusters was selected by simple random sampling from the list maintained by the Department of Census and Statistics, Sri Lanka. The second stage sampling unit consisted of individual households. From each GN, 40 households were sampled, the first household being selected randomly from the electoral register. Proximity selection was then used to select subsequent households as the "next nearest" until the desired sample size was reached. In cases of non-response (no one in the household when the interviewers visited) the house was visited once again before the data collection in the cluster was completed. If there was still no respondent, the next nearest house in the cluster was selected.

Data were collected by trained data collectors using an interviewer administered questionnaire. They were assisted by local field volunteers recruited within each cluster. The respondent of each household was either the head of the household or a responsible adult present in the house. A two part structured questionnaire was developed for data collection. The questionnaire was translated to Sinhala and Tamil and was pre-tested in a GN division not selected for the study within each province. Based on the findings of the pre-testing the questionnaire was fine-tuned prior to use.

In the first phase of data collection the research assistant screened the households for snakebite within the previous 12 months and obtained socio-demographic data from the households. In the second phase, data collection instruments were administered to the households where snakebites were reported within the previous 12 months in order to obtain details of the bite, evidence of significant envenoming, and deaths due to snakebite. Significant envenoming was defined as the presence of local tissue necrosis at the site of bite, presence of neurotoxicity or bleeding manifestations.

Data were double entered into databases created in Epidata software version 3.1 [13]. All discrepancies were corrected by referring to the original data sheets.

#### Socio-demographic and environmental data

The following GN-division-level data were obtained from Department of Census and Statistics, Sri Lanka: population density; percentage of males; percentage of agricultural workers; population mean age; percentage of people who had studied up to or above G.C.E. Advanced Level Examination; percentage of the major ethnic group. Mean income is recorded at district level; for the analysis, the district-level value was attached to each GN division within a given district. A climatic zone [8] was similarly attached to each GN division as a categorical variable.

Island wide data on Normalized Difference Vegetation Index (NDVI) was obtained from the International Research Institute for Climate and Society, Earth Institute, Columbia University (http://iridl.ldeo.columbia.edu/). Island wide data on elevation and land cover were obtained from DIVA-GIS (http://www.diva-gis.org/gdata). Spatial resolution of the downloaded NDVI file was 250 meters and elevation and land cover were 30 arc seconds in each coordinate direction, corresponding to pixels of area 0.8 km^2^ at the equator. The values of NDVI, elevation and land cover at the centroid of each GN division was then used as a GN-division-level explanatory variables.

### Statistical methods

#### Exploratory analysis

Data analysis was performed in Stata version 12 [14] and in the R programming language version 3.2.2 [15]. Population based incidence rates of snakebite by province with 95% confidence intervals were constructed taking into account the cluster sampling technique used. National figures were derived taking into account both the stratification by province and the clustering by GN division. Population based incidence rates were calculated using the “Survey” package in the R programming language [16].

Individual level variables (e.g. age, sex) were considered only for descriptive analysis and were not considered for modelling. The explanatory variables for snakebite incidence modelling included cluster level population density, sex, occupation, age, education, ethnicity, income, elevation, NDVI, climatic zones and land use. Only the spatial variables were considered in modelling of the envenoming bite (i.e. population density, elevation, NDVI, climatic zones and land use), based on an assumption that envenoming is related to the geographical distribution of venomous snakes rather than to human snake interaction.

Generalized linear and generalized additive models were used for exploratory analysis of the number of snakebites and number of envenoming bites among the sampled population in each cluster, ignoring spatial correlation. Generalized additive models were used to identify the patterns of association of explanatory variables and to construct piece-wise linear functions so as to address non-linear associations within parametric generalized linear models (S1 Appendix). Subsequently, the significant variables of the respective piece-wise linear models were then used as explanatory variables in geostatistical modeling.

The standardized residuals of the fitted piece-wise linear models were mapped in a Sri Lankan grid to investigate residual patterns, corresponding to spatial variation in incidence that is not captured by the available explanatory variables. The empirical variogram of cluster-level incidence was used to suggest a geostatistical model for the spatial correlation of adjusted bite incidence (S2 Appendix).

#### Geostatistical modelling and predictive mapping

Model-based geostatistics was used to model snakebite incidence and incidence of envenoming in relation to geographic location, accounting for spatial correlation [17], incorporating as explanatory variables the piece-wise linear functions identified by the exploratory generalized linear modelling. Parameter estimates were obtained by the Monte Carlo maximum likelihood method and passed to plug-in spatial prediction for mapping the results (S3 Appendix). Along with the incidence maps with point estimates, probability contour maps (PCM)s were developed to show the spatial variation in the probability that local incidence does or does not exceed any given incidence threshold. In each PCM, red, green and yellow-orange colours indicate high (at least0.7), low (at most 0.3) and intermediate probabilities of exceeding the given threshold. The PrevMap package version 1.2.2 was used to fit the geostatistical model [18].

Based on the geostatistical model, individual point estimate maps and PCMs were developed to demonstrate incidence of snakebite in Sri Lanka (national snakebite incidence rate: 398 per 100000), and to identify hotspots (incidence more than 500 per 100000) and cold spots (incidence less than 300 per 100000) for snakebites. Similar individual maps were developed to demonstrate incidence of envenoming in Sri Lanka (national snakebite envenoming incidence rate: 151 per 100000), and to identify hotspots (incidence more than 250 per 100000) and cold spots (incidence less than 100 per 100000) for envenoming.

### Ethics statement

Ethical approval for the study was given by the Ethics Review Committee of the Faculty of Medicine, University of Kelaniya. Data collection was done by using an interviewer administered questionnaire and all interviews were conducted after obtaining informed written consent. No animals were used in the study. Permission for conducting the study was obtained from District and Divisional level public administrators before data collection. Grama Niladharis of the sampled GN divisions were informed about the study through the public administration system.

## Results

### Exploratory analysis

**Fig 1:**
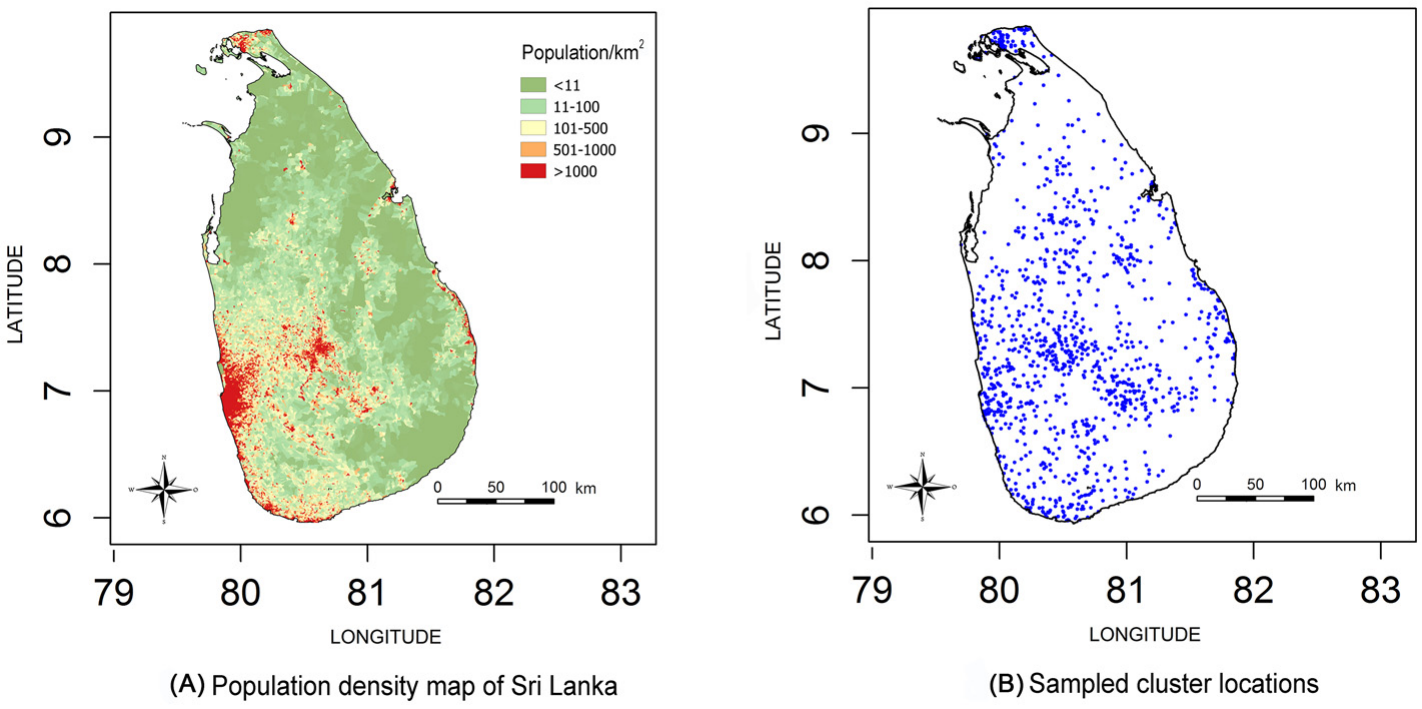
Population density map and sampled cluster locations.

Data relating to 165665 individuals (0.8% of the population of Sri Lanka) living in 44136 households in 1118 clusters were collected (Fig 1). 695 snakebites, 323 envenomings and 5 deaths (four of them male) were reported in the sample population in the 12 months preceding the interview. The incidence of snakebites, envenoming and deaths in the complete sample population was 398 (95% CI 356–441), 151 (95% CI 130–173) and 2.3 (95% CI 0.2–4.4) per 100000 population respectively. Extrapolating this to the population of the whole country, the estimated national numbers of snakebites, envenoming and deaths were 80514 (95% CI 71774–89254), 30543 (95% CI 26203–34883) and 464 (95% CI 45–884) respectively.

There was wide geographical variation in incidence within the country: the Northcentral province (rural, agricultural, dry region) had the highest incidence rate of 623 snakebites per 100000 and the Central province (which includes the high altitude areas of the country) the lowest incidence rate of 277 per 100000 (Table 1). The differences were more marked for envenoming. The Northcentral province had the highest envenoming rate of 440 per 100000 and the Central province the lowest rate of 92 per 100000 (Table 1). The proportion of snakebite victims who were envenomed varied from 20% in Western province to 70% in Northern and Northcentral province.

**Table 1:** Incidence of snakebite and envenoming by province.

Climatic Zone	Province	Bites			Envenomings		
		Reported (sample)	Estimated number	Risk per 100000 (95% CI)	Reported (sample)	Estimated number	Risk per 100000 (95% CI)
Wet	Western	61	18910	325 (217–432)	12	3720	64(027–101)
Wet	Central	45	7065	277(182–371)	15	2355	92 (042–142)
Wet	Sabaragamuwa	102	10506	548 (438–658)	35	3605	188 (127–249)
Wet/Intermediate	Northwestern	92	11776	499(392–605)	34	4352	184(118–251)
Wet/Intermediate	Southern	87	11310	461 (338–584)	18	2340	95 (047–144)
Dry/Intermediate	Uva	63	4095	328 (242–414)	39	2535	203 (137–270)
Dry	Northern	59	3422	324 (219–428)	42	2436	230 (145–316)
Dry	Eastern	67	5695	368 (227–509)	44	3740	242 (119–365)
Dry	Northcentral	119	7735	623 (487–760)	84	5460	440 (325–555)
Total		695	80514	398 (356–441)	323	30543	151 (130–173)

Incidence rates of snakebite and envenoming among males were 478 (95% CI: 415–541) and 176 (95% CI: 146–207) per 100000 population. Males were approximately 1.5 times more likely to experience both snakebites and envenoming than females (Table 2). Median age (interquartile range) of snakebite victims was 42 (31–54) years and for victims with envenoming bites was 42 (30–53). Almost 40% of the snakebites occurred between 4 pm and 8 pm. Snakebite was rarest in the early morning (4 am to 8 am). More than 55% of the male victims were involved in labour-intensive outdoor occupations such as farming and manual labour.

**Table 2:** Reported and estimated snakebites and envenoming bites by sex, age, education, employment and income.

	Snakebite (in sample)	Envenoming bites (in sample)
Sex		
Male n (%)	418 (0.25)	193 (0.12)
Female n (%)	277 (0.17)	130 (0.08)
Age		
Median (IQR)	42 (31–54)	42 (30–53)
	Snakebite incidence [95%CI] (per 100’000 population)	Envenoming incidence [95%CI] (per 100’000 population)
Sex		
Male	478 (414–541)	176 (146–207)
Female	320 (273–367)	126 (100–154)
Education		
No-schooling	509 (281–737)	265 (102–429)
Primary (1–5)	520 (427–613)	234 (178–289)
Secondary (6–11)	458 (400–517)	174 (143–205)
Up to Advance level	286 (219–353)	82 (51–114)
Above Advance level	124 (38–212)	54 (08–100)
Employment		
Field workers	460 (411–509)	197 (167–226)
Others	290 (224–355)	70 (47–94)
Average monthly income (Rupees) (1US$ = SLRs.145)		
< 5000	461 (341–582)	280 (186–375)
5000–10000	436 (353–519)	224 (169–279)
10000–20000	340 (275–406)	157 (116–197)
20000–35000	436 (350–521)	106 (76–136)
>35000	352 (230–474)	100 (43–158)

The commonest clinical feature recalled by the snakebite victims was swelling. Symptoms representing neurotoxicity were reported in 28% of all bites. 37% of the significantly envenomed reported neurotoxic features without bleeding. Bleeding manifestations alone were reported by 8%. Neurotoxicity and bleeding together were reported by 24% (Table 3).

**Table 3:** Clinical features recalled by the snakebite victims (N = 695).

Feature	Number	Percent
Swelling at site of bite	606	87.2
Neurological	197	28.3
Abdominal pain	169	24.3
Tissue necrosis at site of bite	110	15.8
Bleeding manifestations	104	15.0
Renal impairment	33	4.8
**Clinical features recalled by victims with significant envenoming (N = 323)**		
Feature	Number	Percent
Neurological	120	37.2
Bleeding manifestations	27	8.4
Neurological and bleeding manifestations	77	23.8
Other (tissue necrosis, renal impairment)	99	30.6

Exploratory analysis for snakebite incidence using generalized linear models, ignoring spatial dependence, showed population density, elevation, occupation distribution and climatic zones to be significantly associated with incidence, with non-linear effects of elevation and occupation distribution. Elevation, population density and climatic zone were significantly associated with envenoming incidence with a non-linear effect of elevation (S1 Appendix).

### Geostatistical model and predictive maps

According to the geostatistical model, the median predicted incidence for Sri Lanka was 397 per 100000 (IQR: 295 to 515 per 100 000) which is similar to the estimated national snakebite incidence derived from the survey. The fitted geostatistical model for snakebite incidence is summarised in table 4. There was a positive association between elevation and incidence up to 160 meters above sea level, with incidence dropping thereafter. Intermediate and wet climatic zones had higher snakebite incidence compared to the dry zone. Snakebite incidence decreased with increasing population density. Snakebite incidence rapidly increased as the proportion of the population engaged in agriculture increased up to 9%, with a more gradual increase thereafter.

**Table 4:** Parameter estimates from geostatistical model for snakebite incidence.

Variable	Estimate	Std. Error	Z value	P value
(Intercept)	-6.6239	0.13456	-49.2246	<0.001
Elevation	0.0032	0.00079	4.0771	<0.001
Elevation more than 160 meters	-0.0046	0.00085	-5.4389	<0.001
Climate zone 2 (Intermediate)	0.2225	0.10564	2.1068	0.035
Climate zone 3 (Wet)	0.5586	0.11763	4.7492	<0.001
Population density	-0.0002	0.00002	-10.8101	<0.001
Percentage of agricultural workers	7.2235	1.33660	5.4043	<0.001
Percentage of agricultural workers > 9%	-6.4166	1.40170	-4.5778	<0.001
Covariance parameters Matern function (kappa = 0.5)				
sigma^2^	0.189	0.8778		
Phi	0.091	2.5249		
tau^2^	0.304	1.5944		

Table 5 shows the results of the geostatistical model for the incidence of envenoming. There was a positive association with elevation up to 195 meters above sea level, with incidence dropping at higher elevations. The incidence of envenoming was higher in the dry zone compared to intermediate and wet climatic zones and decreased with increasing population density.

**Table 5:** Parameter estimates from geostatistical model for envenoming snakebite incidence.

Variable	Estimate	Std. Error	Z value	P value
(Intercept)	-6.0911	0.05500	-110.7385	<0.001
Elevation	0.0043	0.00049	8.7386	<0.001
Elevation more than 195 meters	-0.0046	0.00053	-8.5696	<0.001
Climate zone 2 (Intermediate)	-0.4781	0.07657	-6.2437	<0.001
Climate zone 3 (Wet)	-0.8093	0.08098	-9.9937	<0.001
Population density	-0.0005	0.00002	-29.0282	<0.001
Covariance parameters Matern function (kappa = 0.5)				
sigma^2^	0.055475	4.4079		
Phi	0.129351	2.4801		
tau^2^	0.247201	5.5109		

**Fig 2:**
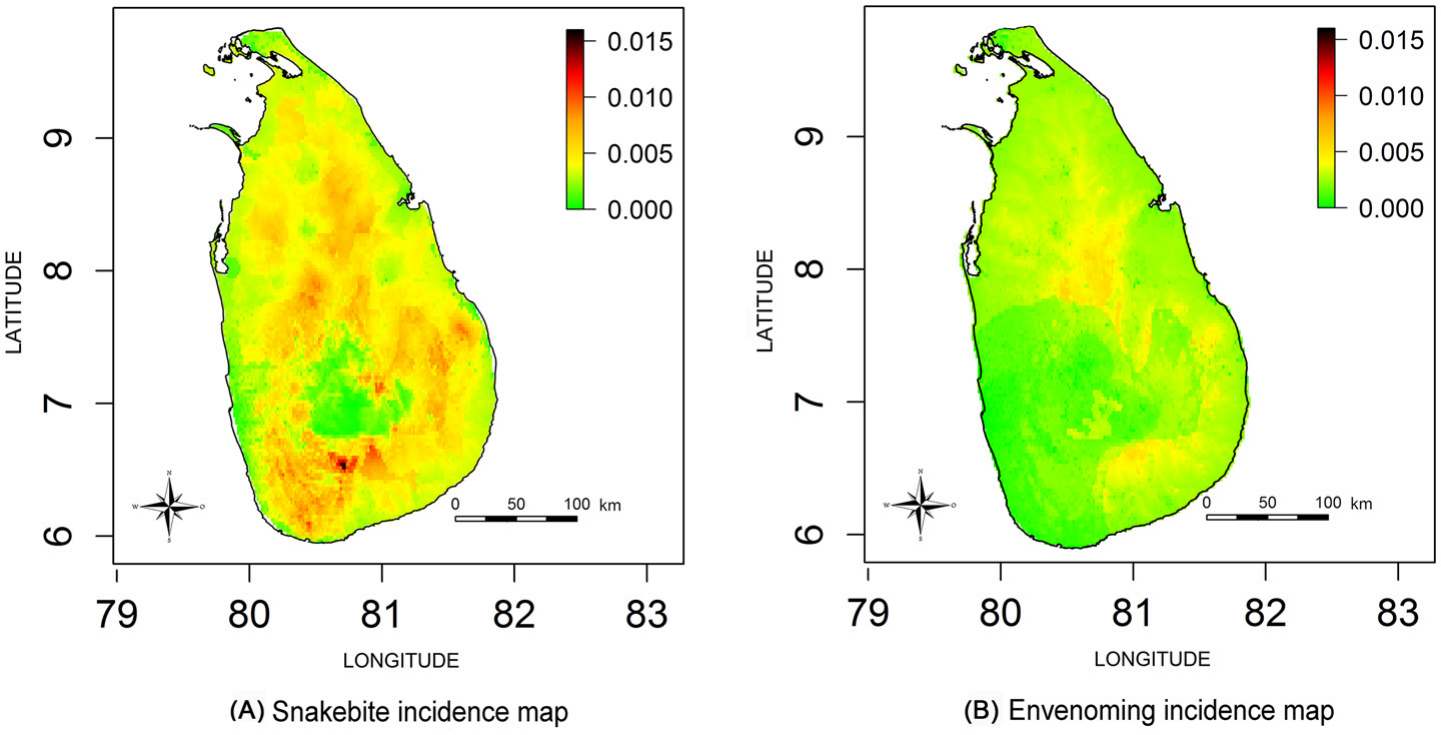
Estimated incidence maps (per 100 cases) for Sri Lanka. (A) Snakebite incidence map. (B) Envenoming incidence map.

**Fig 3:**
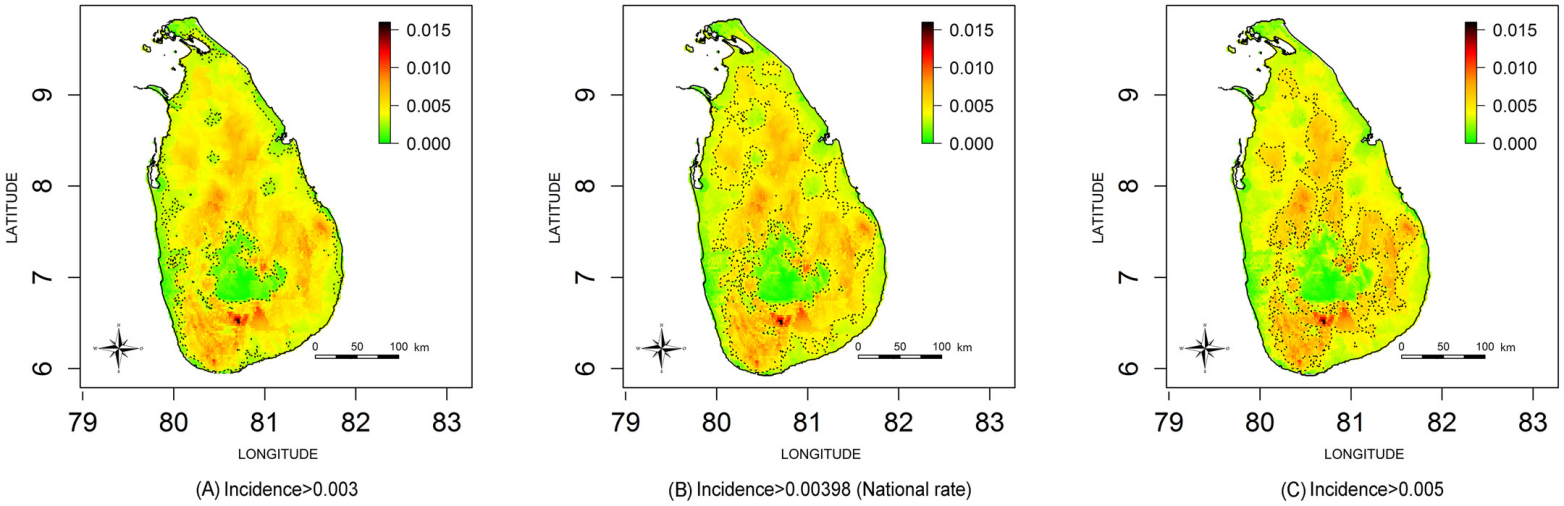
Comparison of snakebite incidence maps for snakebite incidence. Contour lines demarcate incidence higher than (A) 0.003 (B) 0.00398 (i.e. National rate) (C) 0.005.

**Fig 4:**
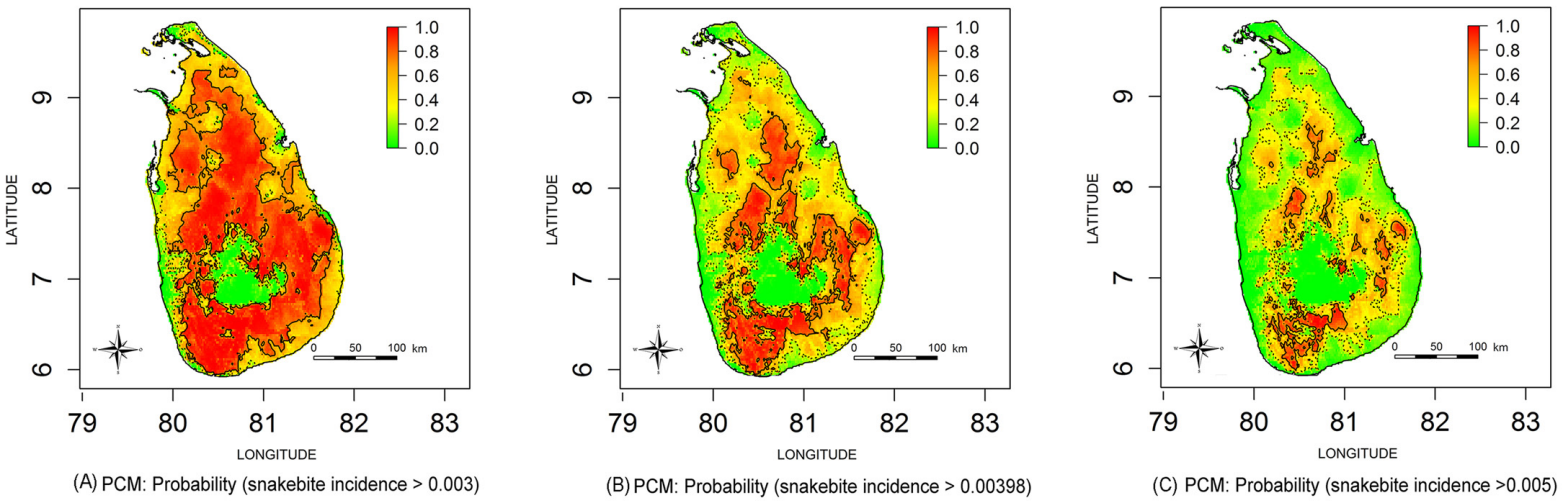
Comparison of probability contour maps (PCMs) for snakebite incidence. pMaps represent probability that snakebite incidence in each area exceeds: (A) 0.003, (B) 0.00398 (i.e. National rate) an (C) 0.005. Contour lines represent P = 0.3 (dash lines) and P = 0.7 (solid lines); green colour area represents the exceedance probability < 0.3 and red colour are represents the exceedance probability > 0.7.

Estimated snakebite incidence and envenoming incidence maps for the whole of Sri Lanka are shown in Fig 2(A) and 2(B) respectively. The three estimated incidence maps shown in Fig 3 include contours demarcating incidence higher than the national snakebite incidence rate (Incidence>398 per 100000) and approximately +/- 100 per 100000 from the national incidence rate for snakebites (i.e. incidence 300 per 100000 and 500 per 100000). Corresponding PCMs based on exceedance probabilities of 300 per 100000, 398 per 100000 (i.e. national rate) and 500 per 100000 are shown in Fig 4. The green areas of Fig 4(A) indicate low probability areas for snakebites (cold spots); in this region the probability of observing a snakebite incidence more than 300 per 100000 is less than 0.3. The red islands of Fig 4(C) show the high probability areas for snakebites (hot spots), where the probability of having a snakebite incidence more than 500 per 100000 is greater than 0.7.

**Fig 5:**
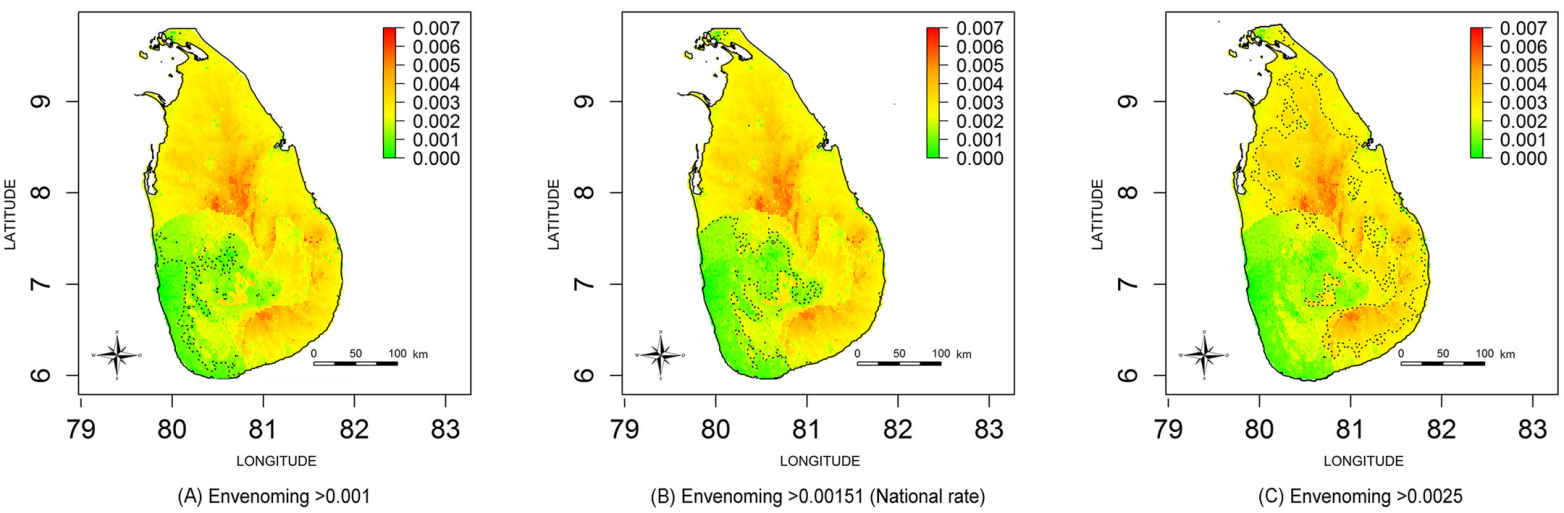
Comparison of envenoming incidence. Contour lines demarcate incidence higher than (A) 0.001, (B) 0.00151(i.e. National rate) and (C) 0.025.

**Fig 6:**
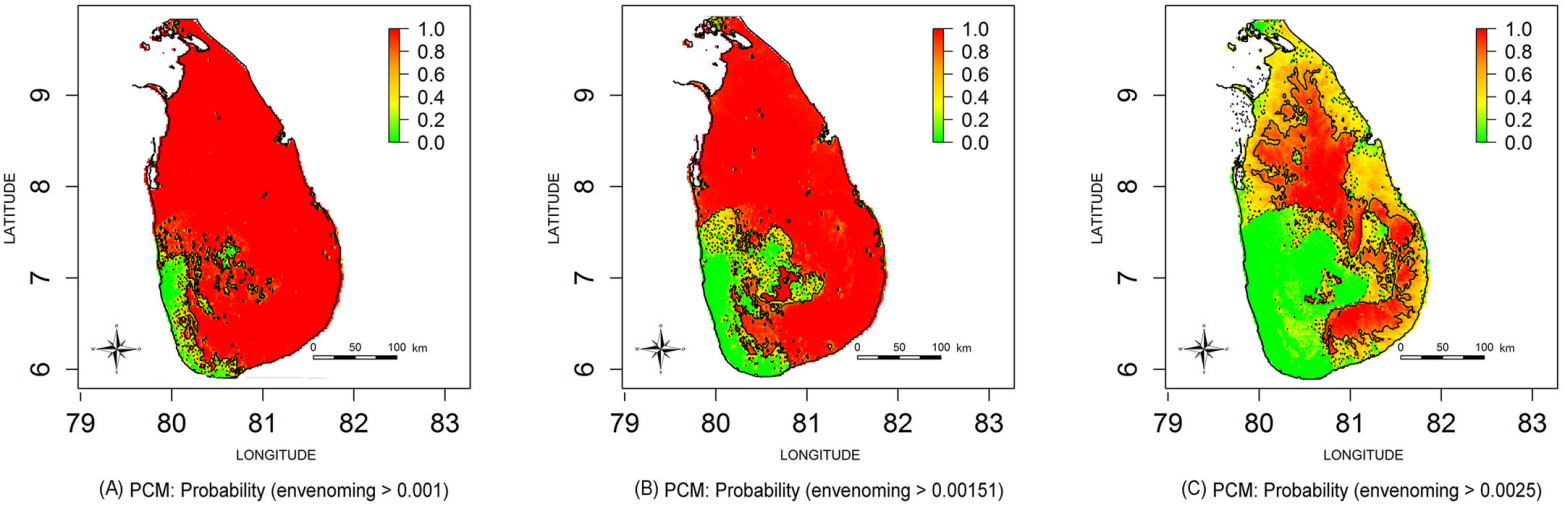
Comparison of probability contour maps for envenoming bites. Maps represents probability that envenoming bite incidence in each area exceeds: (A) 0.001, (B) 0.00151 (i.e. National rate) and (C) 0.025; contour lines represent P = 0.3 (dash lines) and P = 0.7 (solid lines); green colour area represents the exceedance probability < 0.3 and red colour are represents the exceedance probability > 0.7.

The estimated envenoming incidence maps are shown in Fig 5; contours are drawn to demarcate the national rate (151 per 100000), 50 per 100000 lower than the national rate and 100 per 100000 higher than the national rate. PCMs for exceedance thresholds of 100 per 100000, 151 per 100000 (i.e. national rate) and 250 per 100000 are shown in Fig 6(A), 6(B) and 6(C) respectively. The green areas of Fig 6(A) indicate low probability areas for envenoming (cold spots), in this region; the probability of observing a snakebite incidence more than 100 per 100000 is less than 0.3. The red islands of Fig 6(C) shows the high probability areas for snakebites (hot spots); the probability of having a snakebite incidence more than 250 per 100 000 is more than 0.7.

## Discussion

In a nation-wide community-based survey on snakebite we estimated there to be over 80000 bites, 30000 envenomings and 400 deaths per year in Sri Lanka. Our results are supported by a comprehensive methodology that included a representative, large, population-based sample and associated estimation techniques, and represents the most comprehensive survey of snakebite that has ever been undertaken in any country.

Previous studies have attempted to estimate the national incidence of snakebite using hospital-based or local or regional (sub-national) data as highlighted in our estimate of the global burden of snakebite [1]. Such approaches are fraught with inaccuracies as hospital-based data are dependent on health seeking behaviour of the victims and generally underestimate incidence, while localized studies are almost always conducted in regions where snakebite is common, and tend to overestimate incidence [19]. Our previous work has shown that hospital data underestimate deaths due to snakebite by as much as 60% [4]. Incidence data may also depend on seasonal variations of bites. Our study demonstrates comprehensively the considerable geographical variation in snakebite incidence, the variation in the proportion of victims that are envenomed in different regions and the flaws in official data. Consequently, an accurate assessment of the burden of snakebite and envenoming can only be achieved by performing representative community surveys.

Most of our results on the demography of bites are in agreement with the current literature. The highest rates of bites and envenoming were seen in the rural and agricultural north central and north eastern regions of the country. In keeping with other parts of the world, the highest disease burden due to venomous snakebite affects areas with the population groups that are most under-served in terms of healthcare and infrastructure. This re-emphasizes the need for equitable distribution of resources to address the problem of snakebite. Other ecological factors must also be taken into account to better understand these variations. Previous studies on snakebite show that rural males of working age are at high risk of snakebite due to high exposure levels associated with their lifestyle and occupation–mainly farming. In our study, males were also predominantly affected, and for both males and females the most susceptible were the economically active age groups.

We have shown that the incidence of snakebite and envenoming gradually decreases with increasing population density. This may be due to more densely populated areas being less suitable as snake habitats. Snakebite incidence increased with increasing elevation up to 160 meters and thereafter dropped with increasing elevation, likely reflecting the facts that coastal areas are not generally suitable for snake habitats and that temperature declines with increasing altitude.

Agricultural work has commonly been identified as a risk factor for snakebite. We assessed this by using the percentage of all workers undertaking agricultural work in each of the clusters. Snakebite incidence is dramatically lower in non-agricultural communities (less than 9% involved in agriculture) and snakebite incidence increased as the proportion of agricultural workers in a community increased.

The incidence of snakebite is higher in both the intermediate and wet zones than in the dry climatic zone. However, the overall incidence of envenoming is higher in the dry zone. This is likely to reflect variation in species and again shows the importance of capturing data on the incidence of both snakebite and envenoming. Russell’s viper is the most widely distributed snake in Sri Lanka, and can be found up to an elevation of 1800 m. Saw-scaled vipers are largely confined to the arid dry zones of the country including Northern and Eastern Provinces extending up to eastern parts of the Southern Province. Cobras are also widely distributed in the country and are found up to 1500 m. Common Kraits are mainly found in the dry zone. Hump-nosed vipers are found all over the country, but mostly in the wet and intermediate zones [20][21][22][23].

Defining geographical areas by the level of risk of snakebite and envenoming is important for decision makers; allocation and distribution of antivenom, establishing snakebite management centres, and location of emergency treatment units and intensive care units all depend on assessing snakebite risk in a given location. Decision makers require thresholds to identify high risk areas for intervention. In this study, we drew cut-off points based on the national snakebite incidence rate for the country, which is about 400 per 100000. High risk areas or hotspots and low risk populations (cold spots) were defined by adding or subtracting 100 cases per 100000 to the national rate. Similarly, cut-off points were determined for envenoming bites based on the national rate of 150 cases per 100000. Hot and cold spots were determined by adding 100 cases per 100000 and subtracting 50 cases per 100000, respectively, from the national rate.

Such estimates can be further refined to improve decision making by quantifying the likelihoods of exceeding or not exceeding any pre-defined threshold of incidence at a given location. Mapping of estimated incidence may lead to some uncertainty about whether the true incidence exceeds a given cut off threshold, as precision of the estimates varies between locations. Although the point estimate maps and PCMs are qualitatively similar, quantitative differences are important in interpreting the maps and decision making. Thus PCMs provide additional information to identify confidently high risk and low risk areas with respect to any pre-defined incidence threshold, as indicated on a PCM by probabilities close to one or zero respectively. PCMs also identify areas of high uncertainty, i.e. map areas with probabilities close to 0.5, indicating a need for additional sampling to draw firm conclusions.

The methodology used in the present study can be applied to estimate snakebite incidence in other geographical locations. In the methodology, the model captures the complexity of the snakebite process of a human, snake and environment interaction through a combination of covariate adjustments and spatially correlated residual variation, representing explained and unexplained spatial variation, respectively. This flexibility allows the approach to be applied in, with re-estimation of the model parameters to take account of difference in the available covariate information and consequent re-balancing between explained and unexplained spatial variation.

There are inevitably limitations to this study. The quality of the household data depended upon memory of an event and is therefore subject to recall bias. However, the main outcome of interest, a snakebite, is highly memorable and we think it unlikely that this affects our estimates although the possibility of the incidence of envenoming being increased due to inappropriate classification of envenomed bites is acknowledged. Our sampling strategy was carefully designed to make it representative of Sri Lanka. However, the use of the electoral register to identify the first household in any cluster does mean that there is a chance that the small population in slums may have been under-sampled. As slums are relatively uncommon in Sri Lanka except in big cities, this is unlikely to have affected our estimates. Urbanization, which is highly correlated to several of the district-level variables, could also have been a confounder. Finally, cluster (i.e. GN) level income statistics were not available at the Census and Statistics Department of Sri Lanka. We therefore used district mean income to estimate cluster level income. This approach means that variability in income within a district may have led to some inaccuracies in the cluster level income estimates resulting in some residual confounding.

In conclusion we have, for the first time, been able to provide robust community based incidence rates of snakebite and envenoming at a national level. We also developed estimated incidence maps and probability contour maps of within-country spatial variation, which can inform local healthcare decision making. The methodology we have used is replicable, as the required software is open-source. It also addresses the methodological limitations of the many epidemiological studies on snakebite that base their results on data from hospitals or from localized or time-limited surveys.

## Supporting Information

S1 Appendix Generalized linear models for snakebite and envenoming bite incidence. (DOCX)

S2 Appendix Assessing spatial dependence. (DOCX)

S3 Appendix Geostatistical modelling of snakebite and envenoming bite incidence. (DOCX)

S1 Dataset Data. (CSV)
