# Supplementary material for: Mapping the Risk of Snakebite in Sri Lanka - A National Survey with Geospatial Analysis
Source: PLoS Negl Trop Dis. 2016 Jul 8;10(7):e0004813. doi: 10.1371/journal.pntd.0004813 (PMC4938527; doi:10.1371/journal.pntd.0004813)
Supplement: S1 Appendix — (DOCX) [file pntd.0004813.s001.docx]

# S1 Appendix: Generalized linear models for snakebite and envenoming bite incidence

## Snakebite incidence:

Logistic regression modelling assuming no spatial dependence showed population density, elevation, percentage of agricultural workers in the cluster, and climatic zones to be significant explanatory variables for snakebite incidence and this model is considered as “non-spatial logistic model for snakebite incidence” (Table S1.1).

**Table S1.1: Parameter estimates from non-spatial logistic model for snakebite incidence**

|  | Estimate | Std. Error | Z value | P value |
| --- | --- | --- | --- | --- |
| (Intercept) | -6.858 x 100 | 3.091 x 10-01 | -22.187 | < 2 x 10-16 |
| Elevation | 3.994 x 10-02 | 1.247 x 10-02 | 3.202 | 1.364 x 10-03 |
| Climate zone 2 (Intermediate zone) | 3.168 x 10-01 | 1.155 x 10-01 | 2.742 | 6.109 x 10-03 |
| Climate zone 3 (wet zone) | 6.021 x 10-01 | 1.315 x 10-01 | 4.579 | 4.67 x 10-06 |
| Population density | -2.679 x 10-04 | 5.954 x 10-05 | -4.499 | 6.83 x 10-06 |
| Percentage of agricultural workers | 7.166 x 100 | 2.163 x 100 | 3.313 | 9.25 x 10-04 |

Generalized additive models were used to identify the non-linear association of explanatory variables. Elevation and percentage of agricultural workers in the cluster had non-linear distributions and piece-wise function was considered. Knot positions of piece-wise functions for elevation and percentage of agricultural workers in the cluster were determined based on maximising the log likelihood for different knot positions. Initially, piece-wise function was introduced to each of the two variables separately, where piece-wise function was introduced to elevation while assuming linearity for percentage of agricultural workers in the cluster along with the other variables in the non-spatial logistic model and vice versa. Maximum log likelihood was observed with knot placements at 160 meter for elevation piece-wise model and 0.08 for percentage of agricultural workers in the cluster. Both elevation piece-wise model (Log likelihood -1115.309, df=7, delta deviance: 8.318, P=0.0039) and percentage of agricultural workers’ piece-wise model (Log likelihood -1117.868,df=7,delta deviance: 5.759, P=0.0164) showed higher maximised log likelihood compared to a non-spatial logistic model without piece-wise function (i.e. Log likelihood = -1123.627, degrees of freedom=6) (Table S1.2).

However, log-likelihood improvement was more than the above two individual piece-wise models once piece-wise functions were considered for both elevation and percentage of agricultural workers in the cluster simultaneously. This was done by alternating between maximising the log likelihood over different knot positions for elevation with a fixed knot position forpercentage of agricultural workers in the cluster, and similarly maximising for different knot positions in percentage of agricultural workers with a fixed knot position for elevation. The log likelihood was maximized with knot positions at 160m for elevation and 0.09 for percentage of agricultural workers, and the piece-wise model showed a significant improvement in the model fit compared to non-spatial logistic model(delta deviance = 25.9, df=2, P = 2.37e-06); see Table S1.2.

**Table S1.2: Comparison of log-likelihood of models**

| Model | Deviance | DF |
| --- | --- | --- |
| Non-spatial logistic model (without piece-wise function) | -1123.7 | 6 |
| Non-spatial logistic model with piece-wise function for elevation | -1115.3 | 7 |
| Non-spatial logistic model with piece-wise function for occupation | -1117.8 | 7 |
| Non-spatial logistic model with piece-wise functions for elevation and occupation | -1097.8 | 8 |

Parameter estimates for the fitted piece-wise linear model are shown in Table S1.3. Although there was a positive association between elevation and incidence up to 160 m above sea level, incidence dropped thereafter. Similarly, snakebite incidence rapidly increased as farming percentage increased up to 9% and the increase was gradual thereafter. Incidence decreased with increasing population density, and both intermediate and wet zones had higher incidence compared to dry zone.

**Table S1.3: Parameter estimates from piece-wise linear model assuming no spatial dependence for snakebite incidence.**

|  | Estimate | Std. Error | Z value | P value |
| --- | --- | --- | --- | --- |
| (Intercept) | -6.858 x 100 | 3.091 x 10-01 | -22.187 | < 2 x 10-16 |
| Elevation | 3.994 x 10-02 | 1.247 x 10-02 | 3.202 | 1.364 x 10-03 |
| Elevation> 160 m | -4.110 x 10-02 | 1.249 x 10-02 | -3.292 | 9.95 x 10-04 |
| Climate zone 2 (Intermediate zone) | 3.168 x 10-01 | 1.155 x 10-01 | 2.742 | 6.109 x 10-03 |
| Climate zone 3 (wet zone) | 6.021 x 10-01 | 1.315 x 10-01 | 4.579 | 4.67 x 10-06 |
| Population density | -2.679 x 10-04 | 5.954 x 10-05 | -4.499 | 6.83 x 10-06 |
| Percentage of agricultural workers | 7.166 x 100 | 2.163 x 100 | 3.313 | 9.25 x 10-04 |
| Percentage of agricultural workers> 9% | -6.128 x 100 | 2.231 x 100 | -2.746 | 6.026 x 10-03 |

## Envenoming bite incidence

Logistic regression modelling assuming no spatial dependence showed population density resulted in elevation and climatic zones being the significant explanatory variables for envenoming snakebite incidence. This model is considered as the “non-spatial logistic model for envenoming snakebite incidence” (Table S1.4).

**Table S1.4: Parameter estimates from non-spatial logistic model for envenoming bites**

|  | Estimate | Std. Error | Z value | P value |
| --- | --- | --- | --- | --- |
| (Intercept) | -5.609 x 1000 | 8.339 x 10-02 | -67.261 | < 2 x 10-16 |
| Elevation | 2.528 x 10-04 | 2.281 x 10-02 | 1.108 | 2.68 x 10-01 |
| Climate zone 2 (Intermediate zone) | -3.993 x 10-01 | 1.625 x 10-01 | -2.457 | 1.40 x 10-02 |
| Climate zone 3 (wet zone) | -8.218 x 10-01 | 1.702 x 10-01 | -4.829 | 1.37 x 10-06 |
| Population density | -5.067 x 10-04 | 8.776 x 10-05 | -5.774 | 7.76 x 10-09 |

Generalized additive model analysis showed non-linear association between elevation and envenoming bite incidence and a piece-wise function was considered. The knot position of the piece-wise function for elevation was determined by maximising the log likelihood for different knot positions. Maximum log likelihood was observed with knot placement at 195 meter of elevation. This elevation piece-wise model (Log likelihood –705.9275, df=6, Delta deviance: -4.9828, P=0.0256) showed higher maximised log likelihood compared to the non-spatial logistic model (i.e. Log likelihood = -710.9103, degrees of freedom=5). Parameter estimates for the fitted piece-wise linear model are shown in Table S1.5. There was a positive association between elevation and incidence up to 195 meters above sea level, and incidence dropped thereafter. Dry climatic zone had more envenoming bites compared to other two zones and there was a negative association between incidence and population density.

**Table S1.5: Parameter estimates from piece-wise linear model assuming no spatial dependence.**

|  | Estimate | Std. Error | Z value | P value |
| --- | --- | --- | --- | --- |
| (Intercept) | -5.872 x 1000 | 1.211 x 10-01 | -48.493 | < 2 x 10-16 |
| Elevation | 3.609 x 10-03 | 1.088 x 10-03 | 3.318 | 9.08 x 10-04 |
| Elevation> 195 m | -3.885 x 10-03 | 1.230 x 10-03 | -3.160 | 1.580 x 10-03 |
| Climate zone 2 (Intermediate zone) | -5.647 x 10-01 | 1.715 x 10-01 | -3.292 | 9.96 x 10-04 |
| Climate zone 3 (wet zone) | -9.364 x 10-01 | 1.740 x 10-01 | -5.382 | 7.35 x 10-08 |
| Population density | -4.298 x 10-04 | 8.861 x 10-05 | -4.850 | 1.24 x 10-06 |
