# Supplementary material for: Mapping the Risk of Snakebite in Sri Lanka - A National Survey with Geospatial Analysis
Source: PLoS Negl Trop Dis. 2016 Jul 8;10(7):e0004813. doi: 10.1371/journal.pntd.0004813 (PMC4938527; doi:10.1371/journal.pntd.0004813)
Supplement: S2 Appendix — (DOCX) [file pntd.0004813.s002.docx]

# S2 Appendix: Assessing spatial dependence

Cluster level standardized residuals for the fitted piece-wise models were calculated according to the equation S2.1;

Ϯ_i_ =$\frac{y_{i}-{n_{i}p}_{i}}{\sqrt{{{n_{i}p}_{i}(1-p_{i})}}}$ (S2.1)

At location x_i_, y_i_ is the number of snakebites out of n_i_ individuals and p_i_ is the probability that a person at location x will have a snakebite.

Respective maps of the cluster level standardized residuals of fitted piece-wise linear model for snakebite and envenoming bites in Sri Lankan grid showed visually identifiable spatial patterns with respect to cluster locations (Figure S2.1A and S2.1C).

Subsequently, empirical variograms were calculated for snakebite and envenoming bite incidence at cluster level after transforming incidence statistics to empirical logit form (equation S2.2 and S2.3 respectively).

$$logit \left( Snakebite incidence \right) =log\left( \frac{Number bitten+0.5}{Number sampled-Number bitten+0.5} \right) (S2.2)$$

$$logit \left( Envenoming bite incidence \right)=log\left( \frac{Number envenomed+0.5}{Number sampled-Number envenomed+ 0.5} \right) (S2.3)$$

Empirical variograms and theoretical variograms for snakebite incidence and envenoming bite incidence showed the existence of spatial correlation in the incidence data (Figure S2.1 B and S2.1 D respectively).

| 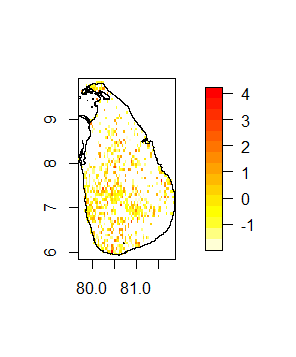   1. Map of standardized residuals at sampled clusters from the non-spatial piece-wise model for snakebite | 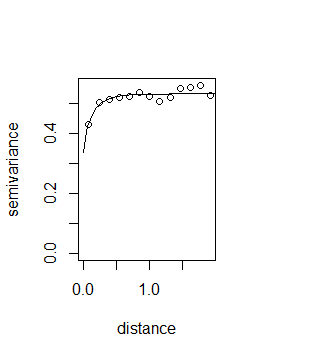   1. Empirical and theoretical variogram for snakebite incidence |
| --- | --- |
| 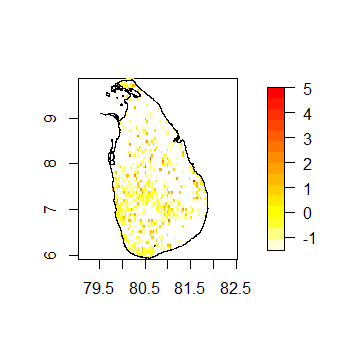   1. Map of standardized residuals at sampled clusters from the non-spatial piece-wise model for envenoming bite | 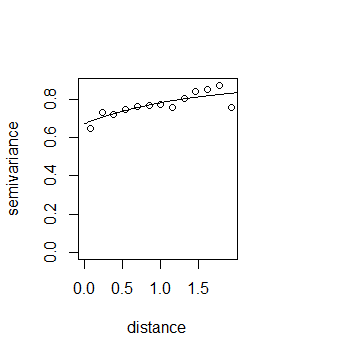   1. Empirical and theoretical variogram for envenoming bite incidence |

**Figure S2.1: Evaluation of spatial correlation: (A) and (C) show standardized residuals are not random at sampled cluster locations for snakebite and envenoming bite incidence respectively (i.e. existence of visually identifiable patterns at sampled clusters). Figure (B) and (D) illustrate the existence of spatial correlation with respect to snakebite and envenoming bite incidence data respectively.**
