# Supplementary material for: Mapping the Risk of Snakebite in Sri Lanka - A National Survey with Geospatial Analysis
Source: PLoS Negl Trop Dis. 2016 Jul 8;10(7):e0004813. doi: 10.1371/journal.pntd.0004813 (PMC4938527; doi:10.1371/journal.pntd.0004813)
Supplement: S3 Appendix — (DOCX) [file pntd.0004813.s003.docx]

# S3 Appendix: Geostatistical modelling of snakebite and envenoming bite incidence

Incidence mapping and spatial predictions were obtained based on the standard geostatistical model (equation S3.1);

Log {p(x)/ [1- p(x)]} = α + f(explanatory variables) + S(x) (S3.1)

At location x_i_, y_i_ is the number of snakebites out of n_i_ individuals. The y_i_ are independent binomial events conditional on an unobserved spatial stochastic process S(x), hence the conditional mean number of snakebite incidence at location x_i_ depends on explanatory variables observed at location x_i_ and on S(x_i_), and p(x) is the probability that a person at location x will have a snakebite. S(x) is modelled as a Gaussian process with mean zero, variance σ^2^ and correlation structure corr[S(x), S(xˊ)]= exp (-u/φ), where u is the distance between x and xˊ and φ is the scale of spatial correlation . The term S(x) in (S3.1) captures the residual spatial variation after adjusting for the covariates.

Separate standard geostatistical models were fitted for snakebite incidence and envenoming bite incidence. Initial values for regression coefficients and covariance parameters were obtained from the respective generalized linear model fit with piece-wise models (i.e. Table S1.3 and Table S1.5) and least-squares estimation of the empirical variogram respectively. A Markov chain Monte Carlo (MCMC) algorithm was used to simulate the samples required for Monte Carlo maximum likelihood estimation. Autocorrelograms and trace plots were assessed to determine the required number of iterations. Series of simulated samples were produced as follows: the numbers of iterations for simulated samples were predefined as 1100, 5000, 10 000, 25 000, 50 000 and 75 000. The first 10% of the samples were discarded as burn-in to minimize dependence on the initial values. Subsequent samples were thinned so as to obtain 1000 samples from the total simulated samples. Thinning reduces the storage requirement and the dependence among sampled values. After accessing autocorrelograms and trace plots (Figure S3.1), 50 000 simulated samples, with 5000 burn in and retention of every 45th sample were selected for parameter estimation and spatial prediction. .

| 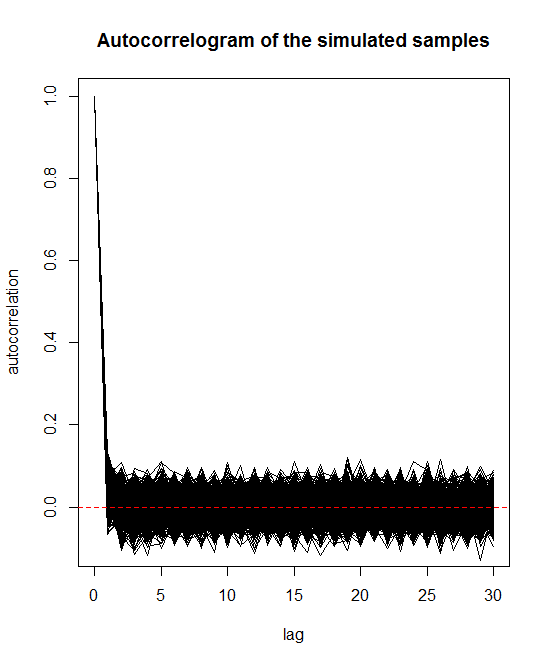 | 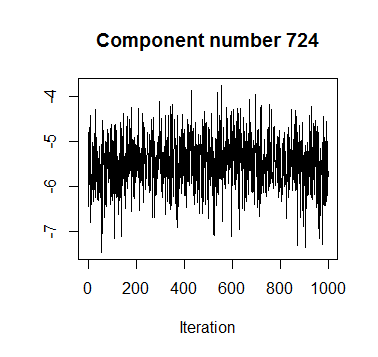 |
| --- | --- |
| (A) Autocorrelogram of the simulated samples for snakebite incidence | (B) Trace plot of the simulated samples for snakebite incidence |
| 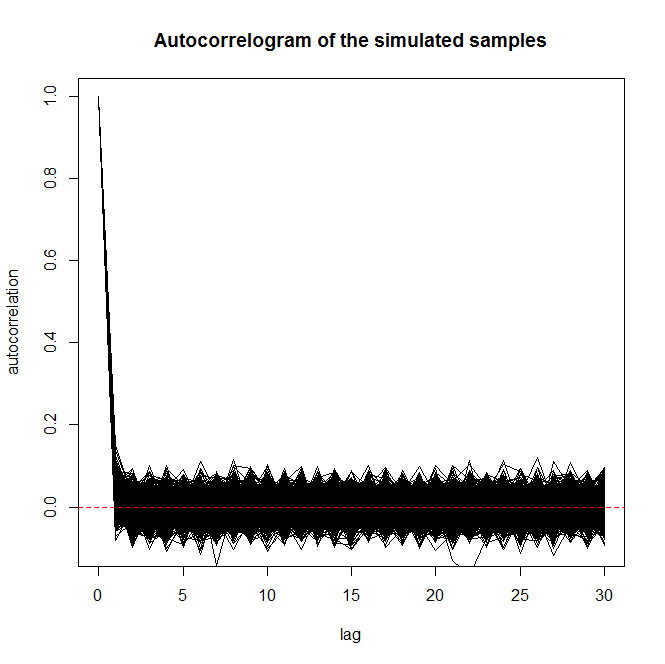 | 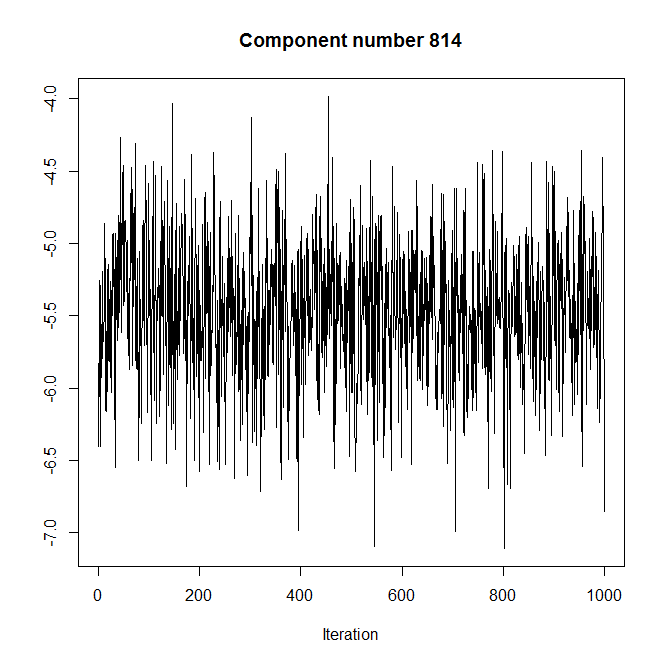 |
| (C) Autocorrelogram of the simulated samples for envenoming bite incidence | (D) Trace plot of the simulated samples for envenoming bite incidence |

**Figure S3.1: Convergence diagnostics: (A) autocorrelogram and (B) trace plot of simulated samples for snakebite incidence; (C) autocorrelagram and (D) traceplot of simulated samples for envenoming bite incidence**

Each required exceedance probability is calculated by first inverting equation (S3.1), whereby S(x) at each sample is converted to a corresponding sample from the predictive distribution of the incidence surface p(x). The corresponding exceedance probability at each location is calculated as the proportion of values of p(x) in a sample that exceeds the specified threshold for p(x).These maps are presented as probability contour maps (PCMs).

Data analysis was performed in the R programming language version 3.2.2 (1).The PrevMap package version 1.2.2 was used to fit the geostatistical model (2).

# References

1. Dessau RB, Pipper CB. R"--project for statistical computing. Ugeskr Laeger. Vienna, Austria; 2008;170(5):328–30.

2. Giorgi E, Peter J. Diggle. PrevMap: Geostatistical Modelling of Spatially Referenced Prevalence Data [Internet]. R package; 2015. Available from: http://cran.r-project.org
